# Personality and Behavioral Predictors of Cyclist Involvement in Crash-Related Conditions

**DOI:** 10.3390/ijerph16244881

**Published:** 2019-12-04

**Authors:** Yubing Zheng, Yang Ma, Nan Li, Jianchuan Cheng

**Affiliations:** School of Transportation, Southeast University, Nanjing 211189, China; mayang93@seu.edu.cn (Y.M.); 220183093@seu.edu.cn (N.L.)

**Keywords:** cyclist, crash risk, risky cycling behavior, personality traits, mediated model

## Abstract

In recent years, the increasing rate of road crashes involving cyclists with a disproportionate overrepresentation in injury statistics has become a major concern in road safety and public health. However, much remains unknown about factors contributing to cyclists’ high crash rates, especially those related to personal characteristics. This study aims to explore the influence of cyclist personality traits and cycling behaviors on their road safety outcomes using a mediated model combining these constructs. A total of 628 cyclists completed an online questionnaire consisting of questions related to cycling anger, impulsiveness, normlessness, sensation seeking, risky cycling behaviors, and involvement in crash-related conditions in the past year. After the psychometric properties of the employed scales were examined, the relationships among the tested constructs were investigated using structural equation modeling. The results showed that cyclists’ crash risks were directly predicted by risky cycling behaviors and cycling anger, and the effects of cycling anger, impulsiveness, as well as normlessness on crash risks, were mediated by cycling behaviors. The current findings provide insight into the importance of personality traits in impacting cycling safety and could facilitate the development of evidence-based prevention and promotion strategies targeting cyclists in China.

## 1. Introduction

### 1.1. Cycling: Risks Outweighs Benefits

Cycling is recognized as an efficient way of improving health and has the potential to tackle obesity, diabetes, and physical inactivity [1,2]. As a mode of transport, cycling is advantaged over motorized transportation in terms of environmental benefits such as zero emissions of greenhouse gas, decreased noise pollution, and reduction of traffic congestion [3]. Given the broad public health benefits, cycling has been promoted extensively worldwide in the last few decades. However, with the growing prevalence of cycling, risks associated with this mode of transportation, especially road crashes involving cyclists, have emerged as a noticeable concern for global public health agencies and practitioners [4].

Cyclists suffer from a heavy burden of road crashes with a disproportionate overrepresentation in traffic injury statistics [5], especially in low and middle-income countries [6]. According to the statistics of World Health Organization [7], pedestrians and cyclists represented 26% of road fatalities all over the world in 2016, with over 40 thousand cyclists falling victim to fatal road crashes just in one year. In the case of China, for instance, in a total of 58,539 road crashes resulting in deaths of road users in 2013, 8% of them involved cyclists [8]. Cyclists are at a higher crash risk than motorized road users (in particular, car drivers) in terms of the number of crashes involved per kilometer or per millionth hour traveled [9,10]. They are also exposed to potentially more serious injuries in collisions due to lack of physical protection and disadvantages of bicycles in mass, speed as well as stability [11,12]. What is even worse, it is well known that crashes involving cyclists are seriously underreported in police, hospital, and insurance claim data [5,13], implying that road safety conditions of cyclists may actually present more threat to public health than it appears.

### 1.2. Factors Related to Crashes Involving Cyclists

Substantial research has been conducted to explore potential causes of the frequent occurrence of cycling-related crashes and factors that may have an impact on crash or injury severities. The three factors that have been widely considered in the literature are cyclists’ age, gender, and exposure to cycling. Generally speaking, older cyclists (particularly those aged over 65) as well as males tend to have a higher crash risk [4,14,15], and cyclists with a greater exposure to cycling (commonly measured by cycling distance, cycling frequency, or cycling time) also have a higher possibility of being involved in crashes [16,17]. Besides, a number of environmental factors regarding the context where the crashes took place, for example, the time of day [18,19] and weather conditions [20], have also been found to be related to cyclists’ road safety outcomes. In addition, prior studies have evaluated the effects of vehicle types, use of protective equipment, and road users’ behavioral patterns on outcomes involving crashes or injuries of cyclists [14,15,21], and certain factors related to infrastructures, roadway design, and traffic control measures have also been identified as stimulus of cycling-related crashes and enhancers of cyclists’ injury severity [22,23,24,25].

Among all the aforementioned factors, the behavior of road users has been proven to be the most immediate determinant of crash involvement [26], with cyclists not being an exception [27]. It is estimated that 60% of the fatal crashes involving cyclists are incurred by their undesirable behaviors such as red-light running, distracted riding, and the occupation of motorized vehicle lanes, and a large proportion of cycling-related crashes involve more than one dangerous cycling behavior [25,28,29]. Martínez-Ruiz et al., [30] noticed that when carrying a passenger or cycling under the influence of alcohol or drugs, the risk of being involved in single-vehicle crashes and collisions with another vehicle was substantially increased. Bacchieri et al., [31] found that the crash risk of commute cyclists engaging in behaviors such as zigzagging through traffic, riding after ingestion of alcohol, and speeding was 1.5 times that of the others. Studies conducted by Useche et al., [21,25] revealed significantly positive effects of cyclists’ traffic violations and riding errors on the number of crashes that they had been involved in. Clearly, risky cycling behaviors not only contribute to the occurrence of traffic accidents but also have an impact on the injury severity of cyclists in collisions [21]. However, compared to the considerable amount of studies which have confirmed the undisputable role of behaviors in automobile drivers’ road safety, research linking cycling behavior with cyclist crashes is relatively scarce. Much remains unknown about the nature of cyclists’ dangerous cycling behaviors, which has hindered the development of interventions and preventive measures specifically targeting cyclist injuries and fatalities.

### 1.3. Personality, Behaviors and Crash Involvement

One of the topics that have been extensively investigated in the research on behavior formation is personality traits. Personality, commonly viewed as an intrinsic characteristic of individual differences, reveals one’s behavioral consistency towards the external environment [32] and has long been cited as a critical precursor of road users’ unsafe behaviors and crash involvement [33,34,35,36]. Interestingly, research examining the direct effects of personality traits on road safety outcomes sometimes revealed a nonsignificant or relatively weak association between these two constructs [32,37], while attempts to consider road users’ behavior as a more distal factor in the personality–crash relationship have been more successful [38,39,40]. The empirical evidence suggests that personality tends to impact crash involvement indirectly through influencing one’s behavior on the road rather than directly contributing to crash risk.

To illuminate the role of personality and behavior in influencing road users’ crash risk, several studies have tried to integrate these constructs into a single structure. For example, Iversen and Rundmo [39] proposed a personality–behavior–crash model and tested it on a sample of Norwegian drivers, and the results showed that risky driving behaviors mediated the effects of drivers’ personality traits on crash involvement. Sümer [40] considered drivers’ dispositional elements and driving behaviors as the distal and proximal context in a contextual mediated model, respectively, and noticed that drivers’ dysfunctional drinking, aberrant driving, and speed choice served as an intermediate construct in the relationship between their crash involvement and psychological symptoms, sensation-seeking, as well as aggression. Similarly, Zhang et al., [36] developed a mediated model combining driving anger, aberrant driving, and crash risk, and found that anger provoked by hostile gestures and arrival-blocking indirectly contributed to drivers’ involvement in adverse outcomes through impacting driving behaviors. Therefore, in light of the available evidence, the role of personality traits should not be neglected when attempting to further understand the mechanisms underlying cyclists’ dangerous behaviors and crash involvement. Nonetheless, research on this issue is still lacking.

### 1.4. The Current Study

In the past few years, the constantly growing number of cyclists worldwide along with their greater exposure to crash risk (i.e., longer time and distance of daily cycling journeys) has led to a substantial rise in crash-related injuries and fatalities in this group, especially in countries where bicycles are used as a common daily mode of transport [21,30]. This situation calls for an urgent need to emphasize issues surrounding cycling safety and simultaneously control the potential risk for global cyclists. The aim of the current study is to explore the role of cyclists’ personality and cycling behaviors in influencing their crash risk. Although there is no standard measure of personality that has been used across studies, personality research of this nature on other types of road users in China can provide some inspiration. For example, road anger and sensation-seeking have been identified as critical precursors to unsafe driving for Chinese drivers [36,41,42]. Research on motorcyclists in China showed that those with a traffic offence history presented a tendency of poor response inhibition [43], and impulsiveness was significantly related to commuter motorcyclists’ ordinary and aggressive violations [44]. Another frequently examined personality trait is normlessness, which has been found to not only add to the endorsement of traffic violations for drivers and e-bikers in China [35,45] but also contribute to Chinese drivers’ involvement in serious and at-fault crashes [35]. Therefore, the four personality traits, road anger (cycling anger in this case), sensation seeking, impulsiveness, and normlessness are adopted in this study.

**Figure 1:**
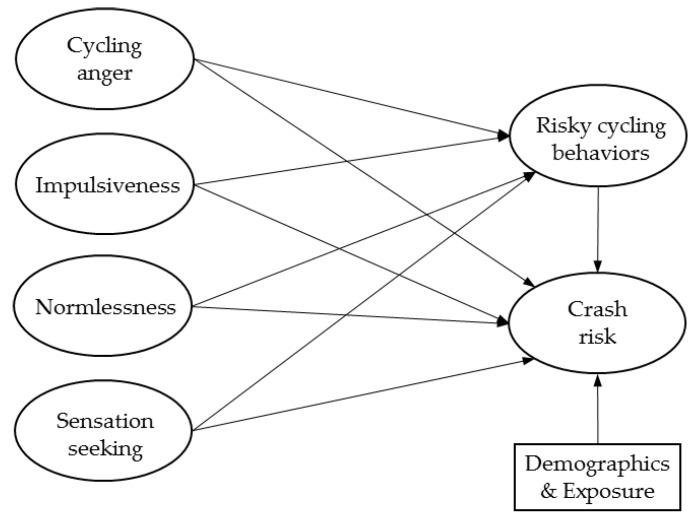
The proposed mediated model.

A model combining the four personality traits, risky cycling behaviors, and cyclists’ crash risk is proposed, as presented in Figure 1. Cyclists’ demographics, as well as cycling exposure, are also included in the model as control variables. We hypothesize that a higher level of cycling anger, sensation seeking, impulsiveness, and normlessness will increase cyclists’ risky cycling behaviors, and risky cycling behaviors are assumed to be positively correlated to cyclists’ crash risk. Besides, the effects of personality traits on crash risk are also presumed to be mediated by cycling behaviors.

## 2. Materials and Methods

### 2.1. Participants and Procedure

This cross-sectional study was conducted with an online survey based on a self-administered questionnaire. The study was approved by the Institutional Review Board of Southeast University Affiliated Zhongda Hospital. The questionnaire was published on one of the largest professional online survey websites in China-Sojump (www.sojump.com). Recruiting efforts included emails, social media, and public forum advertisements. Respondents were considered eligible to participate in the survey if they were no less than 15 years old, were active cyclists (cycled no less than once a month on average), and were able to understand simple statements. Before the survey started, voluntariness, anonymity, and confidentiality of responses were declared to respondents in an electronic consent form, and respondents were encouraged to answer the questionnaire according to their actual thoughts and feelings. It took approximately 15 min to complete the questionnaire. The online survey was opened for 30 days, and the returned questionnaires were carefully examined by two experienced research assistants. There were no missing data in the survey responses, for the online questionnaire could not be submitted with unanswered questions. After excluding questionnaires that were not answered seriously (e.g., choosing the same answer for all questions, answers following a certain tendency, reporting an unreasonable number of crashes), the final sample consisted of 628 participants (364 males and 264 females) with an average age of 31.15 years (S.D. = 9.00 years), and covered 24 provinces and autonomous regions in China.

### 2.2. Measures

#### 2.2.1. Demographic Information and Crash History

Besides the basic demographic information usually surveyed in similar studies (age, gender, and educational background), cyclists were required to report their cycling exposure in terms of cycling frequency and the average weekly cycling distance in the past 12 months. In addition, cyclists were asked about how many times they nearly had an accident (i.e., near-misses), and the number of times they have been involved in minor (those resulting in only material losses or slight personal injuries) and major crashes (those leading to severe personal injuries) during cycling in the past 12 months.

#### 2.2.2. Cycling Anger

Cycling anger is a predisposition to experience anger while cycling, commonly viewed as a situation-specific form of personality trait anger [46]. Respondents’ cycling anger proneness was measured using the cycling anger scale (CAS) [46]. The CAS contains 14 items, each describing one type of cyclists’ problematic interaction with other road users, including police, pedestrians, other cyclists and drivers [40]. Respondents were asked to rate the level of anger they would experience when encountering a given situation during cycling on a five-point Likert scale ranging from 1 (not at all) to 5 (very much). The four-factor CAS showed an acceptable internal consistency, with Cronbach’s *α* ranging from 0.61 to 0.88 for the four subscales and 0.71 for the overall scale [46]. The Chinese version of the CAS was adopted in the current study [47], which had the same factorial structure as the original version and demonstrated a good internal consistency (a Cronbach’s *α* of 0.86 for the entire scale).

#### 2.2.3. Sensation-seeking

Sensation-seeking is defined as one’s willingness to take physical and social risks for a novel experience [48]. The brief sensation-seeking scale (BSSS) [49] was adopted to measure respondents’ sensation-seeking tendency. The BSSS was developed on the widely used sensation-seeking scale [50]. The 8 items covered the four basic aspects of sensation-seeking, including boredom susceptibility, experience-seeking, thrill- and adventure-seeking as well as disinhibition, and the scale revealed satisfactory internal reliability with a Cronbach’s *α* of 0.76 [49]. Respondents were required to rate the extent to which they agreed with each statement on a five-point Likert scale (1 = strongly disagree, 5 = strongly agree). The BSSS has been validated in China by Chen et al., [51], and the Chinese version was used in this study.

#### 2.2.4. Impulsiveness

Impulsiveness is a personality trait addressing one’s tendency to act unguardedly and impulsively, as well as a lack of planning [52]. The Barratt impulsiveness scale-11 (BIS-11) [53] is a commonly used tool to measure an individual’s impulsiveness. The BIS-11 assesses the following three aspects of impulsiveness: motor impulsiveness, attentional impulsiveness, and non-planning impulsiveness. The internal consistency of the scale ranged from 0.79 to 0.83 when tested among different populations [53]. All items of the BIS-11 are answered on a four-point scale (1 = rarely/never, 4 = almost always/ always). A short form of the BIS-11 comprising 15 items (BIS-15) was adopted in this study [54], which retained the three-factor structure as the original version and showed good reliability (Cronbach’s *α* = 0.79) as well as validity.

#### 2.2.5. Normlessness

Normlessness refers to one’s belief that socially unapproved behaviors are necessary to achieve certain goals [55]. Respondents’ normlessness was measured using Kohn and Schooler’s normlessness scale [55]. The scale consisted of 4 items describing one’s belief that deviating behaviors are acceptable to serve for certain goals, which were all answered on a five-point Likert scale ranging from 1 (strongly disagree) to 5 (strongly agree). This study adopted the Chinese version of the normlessness scale, which has been previously applied in Zheng et al.,’s study [45].

#### 2.2.6. Risky Cycling Behaviors

Risky cycling behaviors were examined with the cycling behavior questionnaire (CBQ), an assessment tool specifically designed for cyclists regarding their misbehaviors on the road [56]. In accordance with prior studies [21,25], a combination of two CBQ dimensions, violations and errors, was adopted as the measure of risky cycling behaviors. The violations dimension contains 8 items describing cyclists’ deliberate deviations from practices that are generally considered necessary to maintain the safe operation of bicycles (Cronbach’s *α* = 0.70), and the errors dimension consists of 15 items which depict cyclists’ failure to perform the intended actions in traffic (Cronbach’s *α* = 0.85). Respondents were required to choose how often they performed each type of behavior during cycling in the past 12 months on a five-point Likert scale (1 = almost never/never, 5 = almost always/always).

### 2.3. Statistical Analyses

All analyses were conducted in IBM SPSS v19.0 and the AMOS 21.0 statistical package. First, descriptive statistics were performed on respondents’ demographics, cycling-related experience, and crash history. Second, principal component analyses (PCA) using varimax rotation were applied to examine the factorial structures of the variables. Internal consistency reliabilities of the variables were then assessed with Cronbach’s *α*. In addition, structural equation modeling (SEM) with maximum likelihood estimation was adopted to investigate the validity of the proposed model. A series of indices were selected to evaluate the model fit, including the comparative fit index (CFI), the Tucker–Lewis index (TLI), and the root mean square error of approximation (RMSEA) with a 90% surrounding confidence interval (90% C.I.) [57]. Generally, a value no less than 0.90 for CFI and TLI indicates an acceptable fit of the model to the sample data [57]. The model fit is also judged to be good when RMSEA is no more than 0.06 [57].

## 3. Results

### 3.1. Descriptive Statistics

Descriptive statistics of respondents’ demographics and cycling exposure are presented in Table 1. The respondents ranged in age from 15 to 59, and 78.0% of them were aged between 25 and 44 years. The majority of the respondents (77.2%) had a high school or an undergraduate degree. Only 3.3% of the respondents cycled over 50 km per week, and the percentages of respondents who reported a weekly cycling distance of 11–20 km and 21–50 km were 34.9% and 35.7%, respectively.

In terms of crash history in the past 12 months, the respondents had an average of 0.74 near-miss (S.D. = 0.92) during cycling, and the average numbers of minor and major crashes they have been involved in as a cyclist were 0.36 (S.D. = 0.61) and 0.11 (S.D. = 0.33), respectively.

### 3.2. Factorial Structures and Reliabilities of the Four Personality Traits and Risky Cycling Behaviors

PCA is a widely-used approach to abstract factors from questionnaire responses through summarizing the information included in a set of correlated variables into a smaller number of uncorrelated variables while minimizing the loss of information [58]. A series of PCA with varimax rotation were conducted to investigate whether the original factorial structures of the scales measuring cycling anger, impulsiveness, normlessness, sensation-seeking, and risky cycling behaviors fit the data collected in the present study. The Kaiser criterion (eigenvalues greater than 1.0) [59], the scree plot test [60], and parallel analysis [61] were applied to determine the number of dimensions for each variable.

#### 3.2.1. Psychometric Properties of the CAS

The KMO measure for the CAS items was 0.834, and Bartlett’ test of sphericity was significant at the 0.001 level (chi-square = 3216.73), indicating that the items were well-suited for factor analysis. The PCA yielded four significant factors with eigenvalues greater than 1, and the four-factor structure was then confirmed by the scree plot and the parallel analysis. The first factor included 4 items describing cyclists’ interaction with cars (e.g., “A car forces you off your path”), accounting for 34.68% of the explained variance. The second factor consisted of 4 items measuring cyclists’ anger proneness when interacting with pedestrians (e.g., “Pedestrians are walking on the bicycle lane”), explaining 11.99% of the variance. The third and fourth factor each contained 3 items depicting cyclists’ interaction with police (e.g., “You are fined for cycling on the wrong side of the road”) and other cyclists (e.g., “A cyclist drives very quickly towards you and thereby obstructs you”), and accounted for 10.63% and 8.83% of the variance, respectively. Cumulatively, the four factors explained 66.13% of the variance.

Factor loadings, means, and standard deviations of the CAS items are shown in Table 2. There was no cross-loading for all items, and the factor loadings were all significant and greater than 0.32 (ranging from 0.636 to 0.839), which was generally suggested as the minimum value of an acceptable factor loading [62]. The four-factor structure is in accordance with that found in Zheng et al.’s study [47]. Moreover, Cronbach’s *α* of the whole scale and the four factors ranged between 0.75 and 0.85, indicating a satisfactory internal consistency reliability for the CAS and its four subscales. Therefore, the four-factor solution was used for further analysis, and the score of each factor was represented by the mean score of its subordinate items.

#### 3.2.2. Psychometric Properties of the BIS-15

As for impulsiveness, the 15 items were demonstrated suitable for factor analysis by a KMO measure of 0.859 and a significant statistic of Bartlett’ test of sphericity at the 0.001 level (chi-square = 3913.77). The 15 items loaded on three significant factors with eigenvalues greater than 1. A further examination of the scree plot and the parallel analysis suggested that the three-factor solution could be used for further analysis. Factorial structure, means, and standard deviations of the BIS-15 items are presented in Table 3. The first factor was comprised of 5 items describing one’s control over behaviors and actions (e.g., “I do things without thinking”), and accounted for 38.07% of the variance. The second factor contained 5 items depicting one’s preference to plan ahead for things (e.g., “I plan for the future”), and 11.86% of the variance was explained by the factor. Five items measuring one’s tendency to make quick cognitive decisions (e.g., “I don’t pay attention”) loaded on the third factor and explained 9.81% of the variance. It was noticed that the items “I squirm at plays or lectures” and “Easily bored solving thought problems” cross-loaded on the motor impulsiveness factor and the attentional impulsiveness factor. Therefore, the two items were then eliminated to avoid ambiguity. Factor loadings of the 13 items retained in the final structure were within the range of 0.689–0.801. The three-factor structure also reached a satisfactory internal consistency with Cronbach’s *α* ranging from 0.73 to 0.88 for the subscales and the whole scale. A mean score of each factor was then calculated based on the items within the dimension.

#### 3.2.3. Psychometric Properties of the Normlessness Scale and the BSSS

Appropriateness of the normlessness scale for factor analysis was ensured by the results of the KMO test (KMO = 0.722) and Bartlett’s test of sphericity (chi-square = 308.50, *p* = 0.000). The PCA reproduced a unidimensional structure as in previous studies [45], and the percentage of variance explained by the single-factor solution was 49.04%. Factor loadings of the four items are listed in Table 4 with means and standard deviations. It can be seen that the four items all have significant factor loadings of 0.669–0.736. Besides, the scale also demonstrated acceptable internal consistency with a Cronbach’s *α* of 0.71.

With regards to sensation-seeking, a KMO measure of 0.891 and a significant statistic of Bartlett’s test of sphericity at the 0.001 level (chi-square = 2052.85) showed that the 8 items were suitable for factor analysis. The one-factor solution found in Chen et al.’s study [51] was successfully replicated. Means, standard deviations, and factor loadings of the items are given in Table 5. A total of 53.39% of the variance was explained by the items, of which the factor loadings ranged between 0.614 and 0.815. Cronbach’s *α* of the scale was 0.87, indicating satisfactory internal reliability.

#### 3.2.4. Psychometric Properties of the Scale Measuring Risky Cycling Behaviors

As for the measure of risky cycling behaviors, the KMO measure of the 23 items was 0.962, and the statistic of Bartlett’s test of sphericity was significant at the 0.001 level (chi-square = 6436.84), denoting that the data were appropriate for factor analysis. Two significant factors with eigenvalue over 1 were generated through the PCA procedure and confirmed by the scree plot as well as the parallel analysis. The two-factor solution as well as means and standard deviations of the items are summarized in Table 6. The first factor included 15 items depicting cyclists’ failure to perform planned actions or to achieve certain goals (e.g., “Unintentionally, hitting a parked vehicle”), accounting for 42.15% of the variance. The second factor consisted of 8 items measuring cyclists’ deliberate violation of traffic rules (e.g., “Cycling under the influence of alcohol and/or other drugs or hallucinogens”) and explained 7.52% of the variance. However, 4 items were found to contribute substantially to both factors, for example, the item “Not realizing that a vehicle that was parked intends to leave and hailing to brake abruptly to avoid colliding with it”. The ambiguous items were removed from further analysis. Besides, the two factors both demonstrated good internal consistency reliabilities (Cronbach’s *α* = 0.90 for errors, 0.86 for violations). The mean scores of the two factors were calculated based on the items retained in each dimension.

### 3.3. Relationships among Personality Traits, Risky Cycling Behaviors, and Crash Risk

The proposed model was estimated using SEM. Cycling anger, impulsiveness, normlessness, sensation seeking, and risky cycling behaviors were analyzed as latent variables in the model. The independent variable, crash risk, was denoted by the number of times a respondent had been involved in near-misses, minor and major crashes. Demographics including gender, age, educational level, and weekly cycling distance were included in the model as control variables. Covariance between the four personality variables was also taken into account. Prior to the estimation of the proposed model, indicators of variables measuring personality traits and cycling behaviors were subject to normality tests. As a rule of thumb, the assumption of normality is considered to be met when the skewness and the kurtosis values are within the range of ±2.0 [63]. In this study, absolute values of skewness and kurtosis for these variables were all lower than 1.5 (shown in Table A1), suggesting that the variables were all within the normal range and could be analyzed using SEM.

**Figure 2:**
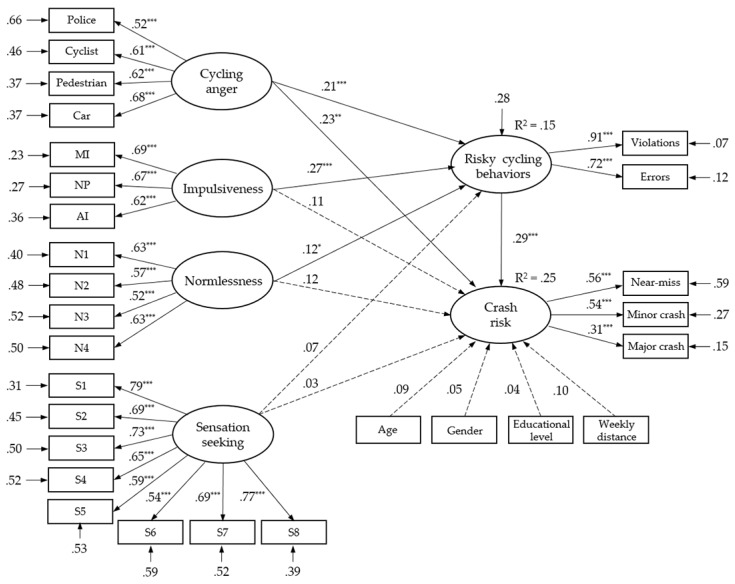
Structural equation model of cyclists’ demographics, personality traits, risky cycling behaviors, and crash risks. Note: The dashed line denotes the nonsignificant path between the two variables connected. *** *p* < 0.001, ** *p* < 0.01, * *p* < 0.05.

Standardized estimates of the hypothesized model are shown in Figure 2. The overall model demonstrated an adequate fit to the sample data: *χ*^2^(335) = 687.674, *p* = 0.000, CFI = 0.933, TLI = 0.924, RMSEA = 0.045 (90% C.I.: 0.039–0.051). Although the *χ*^2^ statistics were significant at the 0.001 level, the *χ*^2^/df ratio was smaller than the acceptable value of 5 [64]. It can be seen that among the four personality traits, cycling anger, impulsiveness, and normlessness were significantly and positively correlated to risky cycling behaviors (*β* = 0.12–0.27, *p* < 0.05), while sensation-seeking showed a nonsignificant relationship with cycling behavior (*β* = 0.07, *p* > 0.05). Impulsiveness emerged as the strongest predictor of risky cycling behaviors (*β* = 0.27, *p* < 0.001). A total of 15 % of the variance in risky cycling behaviors was explained by the four personality traits. Besides, risky cycling behaviors had a significant effect on crash risk (*β* = 0.29, *p* < 0.001), and cycling anger also functioned as a significant predictor of crash risk (*β* = 0.23, *p* < 0.01). That is, cycling anger not only affected crash risk in a direct way but also contributed to crash risk indirectly through impacting cycling behaviors. However, none the other three personality traits (*β* = 0.03–0.12, *p* > 0.05) nor the demographic variables (*β* = 0.04–0.10, *p* > 0.05) had significant direct impacts on crash risk. As a result, a cumulative of 25% of the variance in crash risk was accounted for by these variables.

Furthermore, a bootstrapping procedure was conducted to investigate the magnitude of effects exerted by the four personality traits on crash risk. Indirect effects at the 95% confidence interval were calculated using 5000 bootstrapping samples. The standardized total, direct and indirect effects of the four personality traits, are summarized in Table 7. As shown in the table, sensation-seeking did not reveal a significant direct or indirect effect on crash risk. Cycling anger, impulsiveness, and normlessness exerted significant indirect effects on crash risk through impacting risky cycling behaviors. The summated effect of cycling anger was stronger than that of impulsiveness and normlessness.

## 4. Discussion

This study was driven by the motivation to explore the impact of cyclists’ personality and risky cycling behaviors on road crash risk. The results showed that cycling anger, impulsiveness, and normlessness were significantly and positively correlated to risky cycling behaviors, and the risk of being involved in crash-related conditions was directly predicted by risky cycling behaviors and cycling anger. Moreover, the effects of cycling anger, impulsiveness, and normlessness on crash risk were mediated by cycling behaviors.

The final model revealed a significant correlation between cycling anger and cyclists’ risky riding, indicating that cyclists who are more prone to be provoked in traffic are more likely to violate traffic rules and commit unintentional errors. Although similar relationships have been documented in several earlier studies [46,47], the current results would provide a clearer picture of the association between these two constructs by employing a latent variable structure as well as a more comprehensive measure of cycling behavior. The findings also resemble conclusions drawn by previous research on automobile drivers, where driving anger was found to significantly increase drivers’ endorsement of speeding, ordinary violations, and aberrant driving [39,41,65]. Individuals who are susceptible to road anger tend to show more risk-supportive attitudes [66], and are likely to suffer from impaired performance in risk judgement when experiencing anger feelings [67,68]. In that sense, cyclists’ capability to accurately assess the traffic environment and to detect potential hazards on the road could be weakened by cycling anger, which might stimulate their willingness to take risks and in turn add to the endorsement of risky cycling behaviors.

Besides, cyclists who had a higher level of impulsiveness also reported more risky cycling behaviors. Although lacking direct support from existing research on cyclists, the results coincide with a series of studies that have revealed a significant impact of impulsiveness on drivers’ dangerous behaviors behind the wheel [39,69]. Impulsiveness refers to the tendency of acting without thinking, lacking concern for future events, and having difficulty in maintaining attention [53]. Therefore, cyclists who scored higher on impulsiveness might present an absence of advanced cognition regarding the risk underlying the engagement in traffic violations. Moreover, due to the inability to concentrate consistently on the cycling task and to take proactive maneuvers in case of potential hazards, impulsive cyclists tended to commit more riding errors such as failure to successfully interact with other road users. In addition, cyclists’ normlessness also increased their endorsement in risky riding. Similar results were witnessed in Iversen and Rundmo’s study [39] as well as Yang et al.’s study [35], where normlessness significantly predicted drivers’ involvement in ordinary violations and overspeeding. Individuals with a high level of normlessness seemed to experience lower barriers regarding socially unacceptable behaviors with more willingness to behave in a deviant manner [38,43], which might consequently manifest in cycling when such unsafe behaviors served certain personal goals like saving time or for convenience.

Surprisingly, sensation-seeking did not reveal a significant impact on the involvement in risky cycling behaviors, contradictory to a previous finding that a strong correlation existed between sensation-seeking and drivers’ unsafe driving [39,42,65]. This discrepancy is likely to be due to the variations in sample characteristics between this study and previous ones. More specifically, the present study examined a sample consisting of cyclists within a broad age range (15–59 years), while studies that revealed a significant impact of sensation-seeking commonly focused on age-specific and/or gender-specific motorized transport users (e.g., young male drivers or motorcyclists) [65,66]. Given that sensation-seeking is a personality disposition which tends to increase during childhood to early adolescence while steadily decline into adulthood thereafter [70], the nonsignificant correlation witnessed in this study may be a result of not targeting subjects with extreme high sensation-seeking tendencies (only 8 respondents scored higher than 4 on sensation-seeking). Another possible explanation could be that the risky cycling behaviors examined in this study included not only traffic violations but also cycling errors. Unlike deliberate misbehaviors such as speeding and drunk driving, cycling errors seem to share fewer similarities with the nature of sensation seeking and may not be used to generate excitement. Nevertheless, an examination of the effect of sensation-seeking is worthy of further study.

In agreement with the literature [21,25], risky cycling behaviors were the strongest predictor of cyclists’ crash risk in this study, implying that cyclists who commit more violations and cycling errors have a higher propensity to be involved in adverse outcomes such as near-misses and road crashes. Moreover, cycling anger, one of the personality traits, also directly predicted cyclists’ crash risk when the effects of demographics and cycling behaviors were controlled, consistent with previous studies where road anger has been identified as a proximal construct to crash-related outcomes for cyclists and drivers [39,71]. The results indicate that cycling anger not only increases the occurrence of crash-related circumstances through impelling cyclists to engage in more risk-taking behaviors but also adds to crash risk in a direct manner. In addition, cyclists’ impulsiveness and normlessness also significantly contributed to crash risk via increasing risky riding. The discrepancy regarding the impact pattern of these personality traits could be caused by the dissimilar nature underlying these dispositions and the different ways they interact with one’s information processing, cognition, and decision making. Nevertheless, significant effects of sensation seeking on cyclists’ crash risk were not found in the current study. This result seemed reasonable given that earlier studies have suggested that a sensation-seeker might not necessarily be a risk-taker and would probably perform risky behaviors safely [72,73].

### 4.1. Practical Implications

This study contributes to the current understanding of the mechanism underlying cyclists’ high crash rates through employing a mediated model combining personality traits, cycling behaviors, and crash risk. The current results shed light on the importance of cycling anger, sensation-seeking, and impulsiveness in the causation of cyclists’ unsafe cycling behaviors and involvement in crash-related outcomes. Cyclists at risk are prone to anger in traffic, tend to act without consideration of the consequences, and have low barriers regarding deviating behaviors. Therefore, interventions should be directed to these high-risk populations to prevent their involvement in risky riding. Notably, the findings did not suggest interventions for changing cyclists’ personality, because personality has been proven to be stable during one’s lifespan and thus less malleable by intervening strategies [74,75]. Nonetheless, prior research has suggested that road anger, a more situation-specific predisposition, could be feasibly reduced through short-term and cost-effective groups focusing on self-managed relaxation coping skills or a combination of relaxation and cognitive skills [76]. This would potentially reduce cyclists’ risky riding induced by cycling anger and might, in turn, help to ameliorate the risk of being involved in road crashes and injuries. Furthermore, the results also provide a possibility to identify the at-risk cyclists through a personality assessment. If the high-risk populations can be identified, particularly at a young age, preventive measures can be implemented to prevent them from developing these unsafe cycling habits. Additionally, the findings also indicate that in the framework of interventions specified for cyclists, their behaviors warrant special attention. Empirical evidence showed that cyclists’ knowledge of traffic rules and risk perception abilities could constitute potential targets for tackling unsafe cycling practices [21]. Mass media campaigns and road safety educational programs (perhaps through collaboration between government agencies and companies providing bike-sharing services) could play a role in raising societal awareness of cycling safety issues, and stricter administrative measures directed to cyclists might also help to mitigate risky riding among Chinese cyclists.

### 4.2. Limitations and Future Research

There remain several limitations to be addressed in this study. First, the sole reliance on self-reported measures has inherent limitations such as susceptibility to respondents’ recall bias or social desirability, especially for measures of risky cycling behaviors and crash involvement. Nevertheless, the validity of self-reported data in the road safety domain and the CBQ used in this study has been verified by previous research [56,77,78], and respondents’ independent answering of the questionnaire might also help to alleviate the social desirability bias. It is worth mentioning that although registered data from police and hospitals can provide valuable insight into traffic accidents, a substantial proportion of cycling-related crashes are underreported, in particular, single-bicycle crashes and those not involving severe material losses or personal injuries. For future research, integrated measures based on self-reports and archived data could be implemented to capture more information about cyclists’ crash risks. Second, all the respondents volunteered for this study, and those who were not active cyclists or younger than 15 were excluded from the survey, which might somewhat limit the generalizability of the current findings. Although the age composition and gender ratio of the current sample were generally similar to that reported by several observational studies on Chinese cyclists [79], it is important that future research should disentangle this issue by testing the proposed model in a more socio-demographically diverse sample. Third, two different aspects of cycling behavior, violations and errors, were considered as one factor in the proposed model. The discrepancy between these two constructs needs to be addressed in future studies so as to better comprehend the mechanism underlying each type of behavior and their association with cyclists’ crash risks.

## 5. Conclusions

This study is the first attempt to explain cyclists’ crash risks from a combined perspective of personality and cycling behavior. The results showed that cyclists’ road anger, impulsiveness, and normlessness were positively related to risky cycling behaviors. Cyclists’ crash risks were not only predicted by cycling anger and risky cycling behaviors but also indirectly influenced by cycling anger, impulsiveness, and normlessness through the mediation of cycling behaviors. Cyclists’ demographics and weekly cycling distances did not have an impact on crash risk. The current findings highlight the role of personality traits in impacting cyclists’ cycling behaviors and involvement in crash-related conditions and can aid in developing evidence-based strategies to promote cycling safety in China.

## Figures and Tables

**Table 1:** Participants’ demographics and cycling exposure.

Variable	N	Percentage (%)
Gender		
Male	364	58.0
Female	264	42.0
Age group		
15–24	152	24.2
25–34	272	43.3
35–44	155	24.7
Over 44 years	49	7.8
Educational level		
Middle school and below	94	15.0
High school	248	39.5
Undergraduate degree	237	37.7
Postgraduate degree and over	49	7.8
Weekly cycling distance		
0–10 km	164	26.1
11–20 km	219	34.9
21–50 km	224	35.7
50–100 km	14	2.2
Over 100 km	7	1.1

**Table 2:** Factor loadings, means, and standard deviations of the CAS items.

Items	Factor 1-Car Interaction	Factor 2-Pedestrian Interaction	Factor 3-Police Interaction	Factor 4-Cyclist Interaction	Mean (S.D.)
A fast driving car overtakes you leaving very little space between you.	0.785				3.62 (1.03)
A car overtakes you in a narrow lane.	0.781				3.45 (1.00)
A car forces you off your path.	0.752				3.79 (0.99)
A car fails to give you the right of way.	0.724				3.34 (1.11)
Pedestrians are walking on the bicycle lane.		0.783			3.08 (1.03)
A pedestrian blocks the bicycle lane.		0.783			3.13 (1.01)
A pedestrian surprisingly steps on your bicycle lane in front of you.		0.742			3.36 (1.00)
A pedestrian deliberately blocks your bicycle lane.		0.636			3.77 (0.95)
You are fined for cycling on the wrong side of the road.			0.839		2.79 (1.10)
You are fined for cycling without lights.			0.818		2.90 (1.09)
You are fined as your bicycle is considered not fit for the road.			0.813		3.12 (1.15)
A cyclist drives very quickly towards you and thereby obstructs you.				0.803	3.12 (1.03)
A cyclist overtakes you in a narrow lane.				0.780	2.98 (1.05)
A cyclist forces you off your path.				0.744	3.33 (1.06)
Eigenvalue	4.855	1.678	1.488	1.236	
Explained variance (%)	34.68	11.99	10.63	8.83	
Cronbach’s *α*	0.77	0.85	0.82	0.75	0.85
Mean (S.D.)	3.55 (0.83)	3.33 (0.77)	2.93 (0.95)	3.14 (0.85)	3.27 (0.61)

Note: S.D. = standard deviation.

**Table 3:** Factor loadings, means, and standard deviations of the BIS-15 items.

Items	Factor 1-MI	Factor 2-NP	Factor 3-AI	Mean (S.D.)
I act on impulse.	0.801			1.76 (0.82)
I say things without thinking.	0.739			1.86 (0.82)
I buy things on impulse.	0.735			1.83 (0.84)
I do things without thinking.	0.727			1.85 (0.86)
I act on the spur of the moment.	0.689			1.91 (0.82)
I plan for the future.		0.767		2.09 (0.80)
I plan for job security.		0.752		2.04 (0.96)
I am a careful thinker.		0.727		2.03 (0.88)
I save regularly.		0.725		1.93 (0.95)
I plan tasks carefully.		0.721		2.08 (0.88)
I concentrate easily.			0.743	2.17 (1.02)
I don’t pay attention.			0.742	2.06 (0.93)
I am restless at lectures or talks.			0.724	1.86 (0.88)
^*^ Easily bored solving thought problems.	0.369		0.597	1.94 (0.80)
^*^ I squirm at plays or lectures.	0.395		0.562	2.03 (0.84)
Eigenvalue	5.710	1.779	1.471	
Explained variance (%)	38.07	11.86	9.81	
Cronbach’s *α*	0.85	0.84	0.73	0.88
Mean (S.D.)	1.84 (0.66)	2.03 (0.70)	2.03 (0.76)	1.96 (0.55)

Note: MI denotes motor impulsiveness, NP denotes non-planning, AI denotes attentional impulsiveness. Items marked with * were eliminated from the following analysis. S.D. denotes standard deviation.

**Table 4:** Factor loadings, means, and standard deviations of the Normlessness items.

Items	Factor 1	Means (S.D.)
It is all right to do anything you want as long as you keep out of trouble.	0.736	1.87 (0.81)
It is OK to get around laws and rules as long as you don’t break them directly.	0.707	2.07 (0.85)
Some things can be wrong to do even though it is legal to do it.	0.688	1.93 (0.84)
If something works, it is less important whether it is right or wrong.	0.669	1.89 (0.85)
Eigenvalue	1.962	
Explained variance (%)	49.04	
Cronbach’s *α*	0.71	
Mean (S.D.)		1.94 (0.59)

Note: S.D. = standard deviation.

**Table 5:** Factor loadings, means, and standard deviations of the BSSS items.

	Factor 1	Means (S.D.)
I would do anything as long as it’s exciting and stimulating.	0.815	2.21 (0.91)
Take adventure always makes me happy.	0.794	2.45(0.98)
I’m interested in almost everything that is new.	0.769	2.28 (1.04)
To pursue new stimulus and excitement, I can go against rules and regulations.	0.736	2.32(0.92)
I would love to socialize with adventurous people.	0.735	2.47 (1.00)
I always like to do things that no one else has done before.	0.701	2.46 (0.94)
I will feel very uncomfortable if I stay in the same place for too long.	0.658	2.31 (0.91)
I get restless if I do the same thing a long time.	0.614	2.24(0.91)
Eigenvalue	4.271	
Explained variance (%)	53.39	
Cronbach’s *α*	0.87	
Mean (S.D.)		2.34 (0.70)

Note: S.D. = standard deviation.

**Table 6:** Factor loadings, means, and standard deviations of the items measuring risky cycling behaviors.

Items	Factor 1-Errors	Factor 2-Violations	Means (S.D.)
Unintentionally, hitting a parked vehicle.	0.704		1.89 (0.76)
Trying to brake but not being able to use the brakes properly due to poor hand positioning.	0.694		1.76 (0.75)
Braking very abruptly on a slippery surface.	0.669		1.84 (0.76)
Unintentionally, crossing the street without looking properly, making another vehicle brake to avoid a crash.	0.665		1.81 (0.75)
When you drive on the right, you do not realize that a passenger is getting out of a vehicle or bus and are close to hitting him or her.	0.642		1.88 (0.76)
Fail to notice the presence of pedestrians crossing when turning.	0.639		1.86(0.78)
While you’re distracted, you do not realize that a pedestrian intended to cross a crosswalk, and so you do not stop to let him or her do so.	0.639		1.85(0.72)
Failing to be aware of the road conditions and therefore falling over a bump or hole.	0.638		1.86(0.75)
Brake suddenly and be close to causing an accident.	0.637		1.89(0.80)
Trying to overtake a vehicle that had previously used its indicators to signal that it was going to turn, having to brake.	0.631		1.84(0.76)
* Not realizing that a vehicle that was parked intends to leave and hailing to brake abruptly to avoid colliding with it.	0.616	0.310	1.88 (0.76)
* Colliding (or being close to it) with a pedestrian or another cyclist while cycling distractedly.	0.602	0.303	1.84 (0.73)
* Mistaking one traffic signal for another, and maneuvering according to the latter.	0.600	0.320	1.87 (0.75)
Not braking on a “Stop” or “Yield” sign and being close to colliding with another vehicle or pedestrian.	0.588		1.93 (0.75)
Misjudging a turn and hitting something on the road or being close to losing balance (or falling).	0.584		1.91 (0.76)
* Have a dispute in speed or “race” with another cyclist or driver.	0.321	0.740	1.94 (0.80)
Cycling under the influence of alcohol and /or other drugs or hallucinogens.		0.723	1.97 (0.84)
Handle potentially obstructive objects while riding a bicycle (food, packs, cigarettes…)		0.710	2.08 (0.83)
Zigzagging between vehicles when using a mixed lane.		0.706	2.03 (0.87)
Going against the direction of traffic (wrong way).		0.663	2.01 (0.91)
Crossing what appears to be a clear crossing, even if the traffic light is red.		0.657	2.06 (0.87)
Carry a passenger on your bicycle without it being adapted for such a purpose.		0.650	2.02 (0.84)
Feeling that sometimes I’m going at a higher speed than I should be going at.		0.640	2.08 (0.86)
Eigenvalue	9.695	1.730	
Explained variance (%)	42.15	7.52	
Cronbach’s *α*	0.90	0.86	0.92
Mean (S.D.)	1.86 (0.53)	2.03 (0.63)	1.92 (0.51)

Note: Items marked with * were eliminated from the following analysis. S.D. denotes standard deviation.

**Table 7:** The standardized total, direct, and indirect effects of four personality traits on crash risk.

	Cycling Anger	Impulsiveness	Normlessness	Sensation-Seeking
Indirect effects	0.059 **	0.079 **	0.034 *	0.021
Direct effects	0.229 **	0.112	0.116	0.025
Total effects	0.288 **	0.190 *	0.150 *	0.046

Note: ** *p* < 0.01, * *p* < 0.05.

## Appendix A

**Table A1:** Skewness and kurtosis of indicators of the examined variables.

	Skewness	Kurtosis
Cycling anger		
Police interaction	0.058	−0.677
Cyclist interaction	−0.116	−0.800
Car interaction	−0.352	−0.529
Pedestrian interaction	−0.162	−0.459
Impulsiveness		
Motor impulsiveness	1.286	0.779
Non-planning	0.997	−0.072
Attentional impulsiveness	0.513	−0.637
Sensation seeking		
S1	0.533	0.049
S2	0.545	0.102
S3	0.644	−0.054
S4	0.397	−0.156
S5	0.593	0.277
S6	0.417	−0.194
S7	0.425	−0.268
S8	0.572	0.079
Normlessness		
N1	0.864	1.034
N2	0.689	0.669
N3	0.904	0.905
N4	1.007	1.493
Risky cycling behaviors		
Violations	0.488	−0.382
Errors	0.610	0.195
